# Clinical Efficacy and Safety of BIC/TAF/FTC in Elderly HIV‐Infected Individuals in Southwest China: A Retrospective Observation Study

**DOI:** 10.1002/iid3.70264

**Published:** 2025-11-25

**Authors:** Shujing Ma, Xiaoxin Xie, Yanhua Fu, Lin Gan, Xiaoyan Yang, Qing Wang, Hai Long

**Affiliations:** ^1^ Infection Department Guiyang Public Health Clinical Center Guiyang Guizhou China

**Keywords:** B/F/TAF, efficacy and safety, elderly patients with HIV/AIDS

## Abstract

**Background:**

Long‐term outcome data from real‐world studies of the bictegravir emtricitabine tenofovir alafenamide fumarate (B/F/TAF) regimen in the treatment of elderly patients with HIV/AIDS are still limited. This study evaluated the real‐world effectiveness and safety of B/F/TAF in elderly HIV‐infected individuals in southwest China.

**Methods:**

This was an observational, single‐center, retrospective study that enrolled antiretroviral therapy (ART)‐naïve (*n* = 149) and ART‐experienced (*n* = 143) patients with HIV infection between January 2021 and April 2024. The main endpoint was the viral suppression rate of HIV RNA < 50 copies/ml at 48 weeks, and change in CD4 cell count, CD4/CD8 ratio, body weight, blood lipid and safety were secondary endpoints.

**Results:**

The proportions of ART‐naïve and ART‐experienced HIV‐infected cases with VL < 50 copies/mL at 48 weeks were 93.1% and 92.9%, respectively. CD4 cell counts and CD4/CD8 ratios increased significantly from baseline at 48 weeks (*p* < 0.001). In the treatment‐naive group, ALT, AST, eGFR, and Glu decreased significantly from baseline at 48 weeks, while body weight, Scr, TC, HDL, and LDL increased significantly. Among patients previously administered ART, eGFR increased significantly from baseline at 48 weeks, while AST, LDL, and Scr decreased significantly; other indicators showed no significant changes from baseline. The incidence rates of adverse events were 11.7% and 4.3% in treatment‐naïve and treatment‐experienced, respectively.

**Conclusions:**

For elderly HIV/AIDS patients, B/F/TAF is a safe option to achieve and maintain virological suppression and immune recovery. In terms of lipid metabolism, the metabolic effects of BIC/FTC/TAF in the treated patients are not significant, and the effects in untreated patients require longer follow‐up.

## Graphical abstract

This study aims to statistically analyze the clinical effectiveness and safety of BIC/FTC/TAF in elderly HIV/AIDS patients in real‐world settings, providing clinical data to optimize ART regimens for this population. We believe that our research has made a great contribution to the literature, because we found that the BIC/FTC/TAF showed good virological efficacy in elderly patients with many comorbidities and heavy drug burden.

## 1 Introduction

Acquired Immunodeficiency Syndrome (AIDS) is an infectious disease caused by the Human Immunodeficiency Virus (HIV). HIV increases the risk of opportunistic infections and cancer. The most crucial mechanism underlying this condition is immunodeficiency, which is directly attributed to HIV. The emergence of antiretroviral therapy (ART) has reduced complications and new HIV infections and improved the survival rate of patients [1]. The WHO defines patients aged 50 years and older as elderly HIV/AIDS patients [2], with the aging of the global population, the number and proportion of reported HIV/AIDS cases among the elderly in China are steadily increasing. By the end of 2018 [3], a total of 861,042 existing HIV/AIDS cases were reported in China, among whom elderly HIV/AIDS patients accounted for more than 20% of the total number [4].

As the lifespan of people living with HIV (PLWH) continues to increase, it is anticipated that the incidence of medical complications, including cardiovascular diseases, renal dysfunction, cancer, and bone diseases, will also rise [5, 25]. Compared to young PLWH, elderly individuals in China exhibit a higher proportion of late detection, coupled with reduced immunity and poor treatment compliance. Consequently, HIV/AIDS treatment outcomes in this population are suboptimal, resulting in a higher mortality rate [6]. Studies have shown that by 2030, 36% of elderly patients are expected to have two or more complications [7], and non‐HIV‐related chronic diseases may increase with age. The increasing complexity of complications and multidrug treatment in elderly PLWH often leads to more frequent drug‐drug interactions (DDIs). Consequently, the difficulty of ART application and the risk of treatment failure may be heightened.

Bictegravir (BIC) is a novel integrase strand transfer inhibitor (INSTI) that has been formulated into a single‐tablet regimen with emtricitabine (FTC) and tenofovir alafenamide fumarate (TAF), which is recommended to treat adult HIV‐1 infection. The safety and effectiveness of B/F/TAF in the treatment of elderly patients were confirmed in European population [8]. However, the persistence, impact on viral immunology, clinical and metabolic parameters and real data of B/F/TAF as the initial scheme in Asian cases aged 50 and over are still scarce [9]. As a developing country, China has limited access to integrase inhibitors; with most treatments being freely available medications, EFV + 3TC + TDF is the primary regimen, which is used by over 80% of PLWH [10, 11]. Since the inclusion of B/F/TAF in health insurance, an increasing number of patients have selected this drug as their antiviral treatment option. However, data on the efficacy and safety of integrase inhibitors in elderly patients in China are severely lacking, especially with respect to the incidence rates of metabolic and drug adverse events in the real world. Given that the metabolic characteristics of elderly HIV patients differ from those of younger counterparts, this study aimed to explore the clinical effectiveness and safety of B/F/TAF in real‐world settings for elderly HIV/AIDS patients, providing data support for selecting B/F/TAF reverse transcriptase inhibitor therapy.

## 2 Methods

### 2.1 Study Design and Participants

This single‐center, retrospective cohort study was conducted at the Guiyang Public Health Clinical Center, one of the largest southwest infectious disease hospitals in China, which is responsible for treating 20% of patients with HIV infection in Guizhou Province and 80% in Guiyang City. The study recruited 149 ART‐naïve and 143 ART‐experienced elderly PLWHIV who were treated with B/F/TAF for any reason from July 2021 to August 2023. Data were collected up to August 2024.

Inclusion criteria were: (1) age > 50 years and confirmed HIV‐1 infection; (2) initial treatment with the ART regimen of B/F/TAF or conversion to B/F/TAF for any reason. Exclusion criteria were: (1) pregnancy; (2) loss to follow‐up or death; (3) severe liver and kidney dysfunction; (4) missing HIV viral load, CD4 count, CD4/CD8 ratio, and blood biochemistry data at baseline or 48 ± 4 weeks.

### 2.2 Study Endpoints

The primary endpoint was virological suppression rate, defined by the proportion of patients with HIV‐1 RNA < 50 copies/mL after 48 weeks of treatment. Secondary endpoints included CD4 cell count, changes in CD4/CD8 ratio, safety (i.e., adverse events), and changes in metabolic, liver, and renal parameters at 48 weeks of treatment.

### 2.3 Definitions

Virological suppression (VS) was defined as plasma HIV‐RNA viral load (pVL) < 50 copies/mL [12]. Baseline was the first day of ART. Dyslipidemia was defined as high total cholesterol (TC ≥ 5.2 mmol/L), high triglyceride (TG ≥ 1.7 mmol/L), low high‐density lipoprotein cholesterol (HDL‐C < 1.0 mmol/L), or high low‐density lipoprotein cholesterol (LDL‐C ≥ 3.4 mmol/L). Dyslipidemia was present if any one of these four lipid parameters was abnormal [13].

### 2.4 Statistical Analysis

Statistical analyses were performed using SPSS 25.0 (IBM, Armonk, NY, USA). Continuous variables were presented as mean ± standard deviation and compared by the Student's *t*‐test (when normally distributed) or non‐parametric Mann–Whitney *U*‐test (when non‐normally distributed). Categorical data were analyzed by the chi‐squared test and Fisher's exact test. *p* < 0.05 was considered significant.

## 3 Results

### 3.1 Participant Characteristics

**Figure 1:**
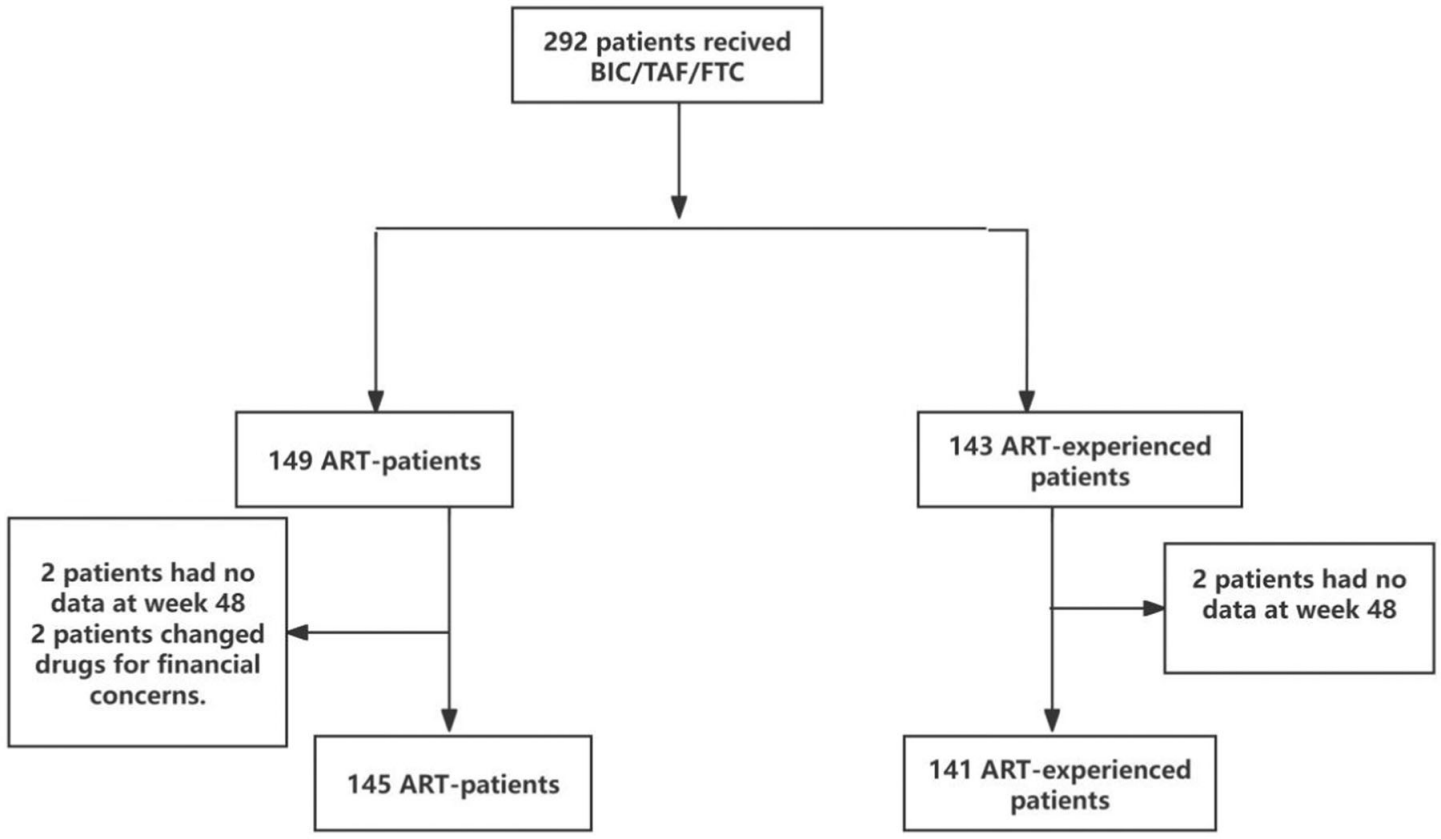
Patient flow chart.

The patient selection process is shown in Figure 1. From July 2021 to August 2024, a total of 292 elderly patients underwent treatment with the B/F/TAF regimen, including 149 newly diagnosed and 143 previously treated patients. Among the newly diagnosed patients, 2 did not complete the 48‐week follow‐up, 2 switched medications due to financial reasons, and 145 completed the 48‐week follow‐up. Among the previously treated patients, 2 had no 48‐week follow‐up data and 141 completed the 48‐week follow‐up.

The ART‐experienced group was predominantly male (74.1%), with a median age of 61.0 years (IQR: 56.0–68.0). At baseline, 88.8% of patients were virologically suppressed (HIV‐VL < 50 copies/mL), and the median CD4+ count was 333.0 cells/μL (range, 242.0–498.5 cells/μL). The patients primarily switched from non‐nucleoside drugs (64.3%), and the main reasons for using BIC/FTC/TAF included renal insufficiency (24.5%) and reduced drug burden (34.2%). Over 44.0% of patients had one or more concomitant medications.

The ART‐naïve group was also predominantly male (61.1%), with a median age of 61.0 years (IQR: 56.0–68.0) years. At baseline, heterosexual transmission was the main mode of transmission (91.1%). CD4 counts were < 200 cells/µL, and 25.5% of ART‐naïve had a viral load higher than 500,000 copies/mL. Totally 34.9% of patients had AIDS‐related opportunist infections, and the main comorbidities in elderly patients were hypertension (15.4%), cardiovascular disease (9.4%) and diabetes mellitus (9.4%). More than 53.7% of patients took one or more drugs (Table 1).

**Table 1:** Baseline characteristics of the included patients.

Characteristic	ART‐naïve (*N* = 149)	ART‐experienced (*N* = 143)
Sex, *n* (%)		
Male	91 (61.1%)	106 (74.1%)
Female	58 (38.9%)	37 (25.9%)
Age median (IQR), year	61.0 (56.0, 68.0)	61.0 (56.0, 68.0)
CD4 count		
Median (IQR), cells/L	297 (120.8, 442.3)	333.0 (242.0, 498.5)
< 200 cells/µL, *n* (%)	66 (44.3%)	22 (15.4%)
≥ 200 cells/µL, *n* (%)	83 (55.7%)	121 (84.6%)
HIV viral load, copies/mL, *n* (%)		
< 50	0	127 (88.9%)
50–499,999	111 (74.5%)	14 (9.7%)
≥ 500,000	38 (25.5%)	2 (1.4%)
Comorbidities, *n* (%)		
Cardiovascular disease	14 (9.4%)	12 (8.4%)
Diabetes	14 (9.4%)	11 (7.7%)
Hypertension	23 (15.4%)	17 (11.9%)
HBV	7 (4.7%)	11 (7.7%)
Osteopenia	7 (4.7%)	13 (9.1%)
Renal impairment	19 (12.8%)	35 (24.5%)
AIDS‐related opportunistic infections, *n* (%)	52 (34.9%)	6 (4.2%)
Previous ART, *n* (%)		
2NRTI + NNRTI	NA	92 (64.3%)
2NRTI + INSTI	NA	39 (27.3%)
Other schemes	NA	12 (8.4%)
Reasons for treatment change		
Renal impairment	NA	39 (27.3%)
Drug resistance	NA	3 (2.1%)
Optimized treatment	NA	49 (34.3%)
Others	NA	52 (36.4%)
Combined medication		
No	69 (46.3%)	80 (55.9%)
Yes	80 (53.7%)	63 (44.1%)

Abbreviations: AIDS, acquired immunodeficiency syndrome; ART, antiretroviral treatment; HBV, hepatitis BINSTIs, integrase inhibitors; IQR, interquartile range; NA, not applicable; NNRTIs, nonnucleoside reverse transcriptase inhibitors; NRTIs, nucleoside reverse transcriptase inhibitors.

### 3.2 Effectiveness

**Figure 2:**
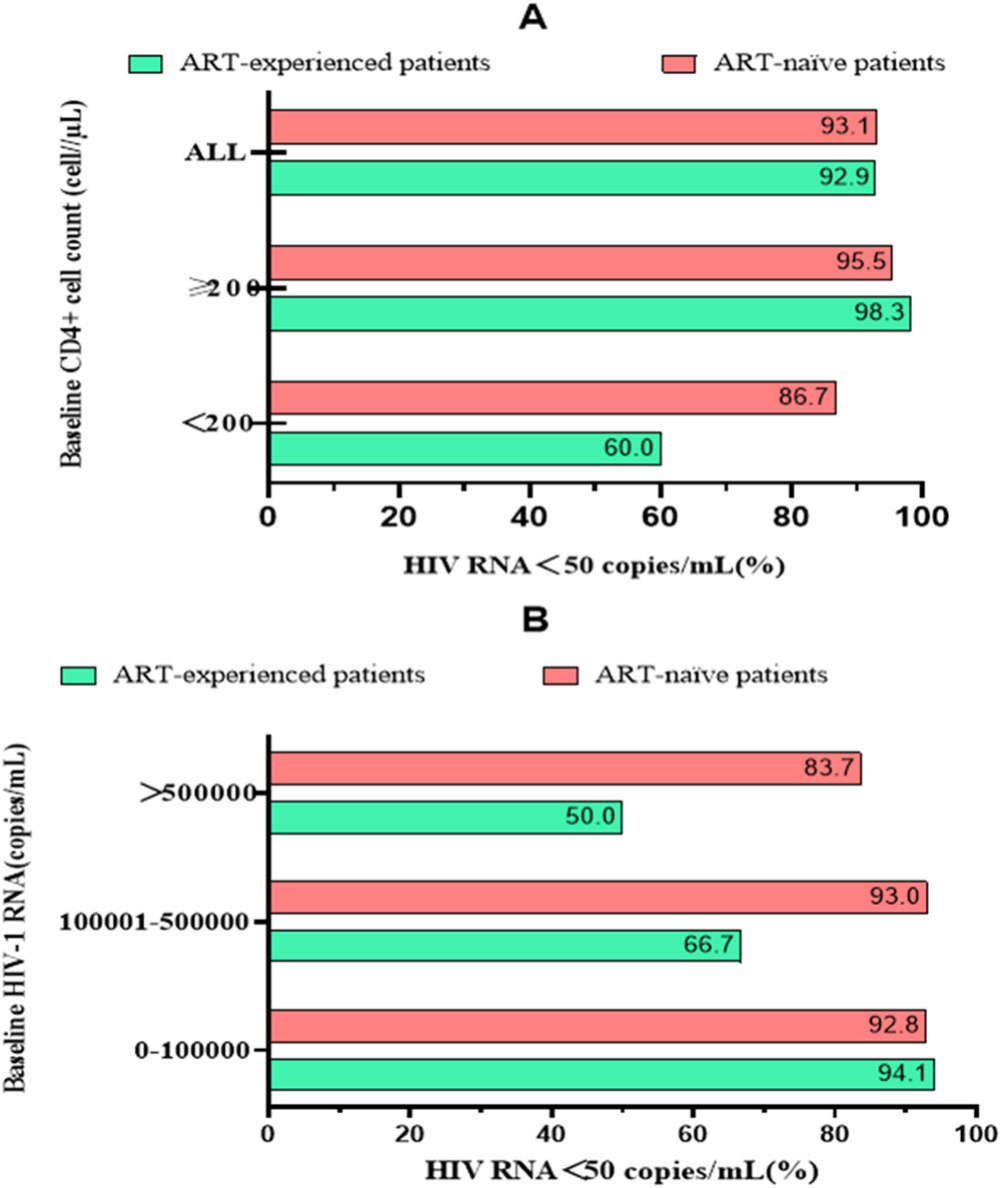
Virological suppression at 48 weeks. (A) Virological suppression rates for different baseline viral loads at 48 weeks. (B) Virological suppression rates for different baseline CD4+ cell counts at 48 weeks.

In the ART‐experienced group, the virological suppression rate at week 48 was 92.9% (131/141). We next stratified these data by baseline CD4 cell count. For participants with baseline CD4 < 200 cells/μL and CD4 ≥ 200 cells/μL, virological suppression rates at 48 weeks were 85% (17/20) and 94.2% (114/121), respectively (Figure 2A). Of the 145 patients initially treated with antiretroviral therapy, 135 (93.1%) had a viral load < 50 copies/mL at week 48. Stratified by CD4 cell count, virological suppression rates for participants with baseline CD4 < 200 cells/μL and CD4 ≥ 200 cells/μL were 86.7% and 95.5%, respectively. For cases with baseline HIV viral load < 10,000 copies/mL and 100,000–500,000 copies/mL, virological suppression rates at week 48 were 92.8% and 93.0%, respectively. In patients with viral load ≥ 500,000 copies/mL, the virological suppression rate at week 48 was 83.7% (31/37) (Figure 2B).

### 3.3 Immunological Changes

Among the 145 ART‐naïve patients, the median CD4 count increased from 180 (IQR: 78.0–314.5) at baseline to 348 (IQR: 280.0–449.5) at 48 weeks, indicating the CD4 count significantly increased by 102.0 cells/μL (*p* < 0.001). The median CD4 + /CD8+ cell ratio also increased from 0.25 (IQR: 0.15–0.43) at baseline to 0.44 (IQR: 0.28–0.76) at 48 weeks, suggesting the CD4 + /CD8+ cell ratio increased by 0.15 (*p* < 0.001). Among the 141 ART‐experienced patients, the median CD4 count increased from 333 (IQR: 242.0–498.5) at baseline to 402 (IQR: 278.0–566.5) at 48 weeks, suggesting the CD4 count significantly increased by 51.0 cells/μL (*p* < 0.001). The median CD4 + /CD8+ cell ratio also increased from 0.62 (IQR: 0.41–0.95) at baseline to 0.71 (IQR: 0.46–1.04) at 48 weeks, indicating the CD4 + /CD8+ cell ratio increased by 0.06 (*p* < 0.001) (Table 2).

**Table 2:** Changes in CD4 cell counts and CD4 + /CD8+ cell ratios at Week 48.

	ART‐naïve (145)	ART‐experienced (141)	*p* value
CD4 count (cells/μL)			
Baseline	180 (78.0, 314.5)	333 (242.0, 498.5)	< 0.001*
At week 48	280 (175.5, 449.5)	402 (278.0, 566.5)	
CD4 + /CD8+ cell ratio			
Baseline	0.25 (0.15, 0.43)	0.62 (0.41, 0.95)	< 0.001*
At week 48	0.44 (0.28, 0.76)	0.71 (0.46, 1.04)	

*Data are presented as median (interquartile range).

### 3.4 Biological Outcomes

**Figure 3:**
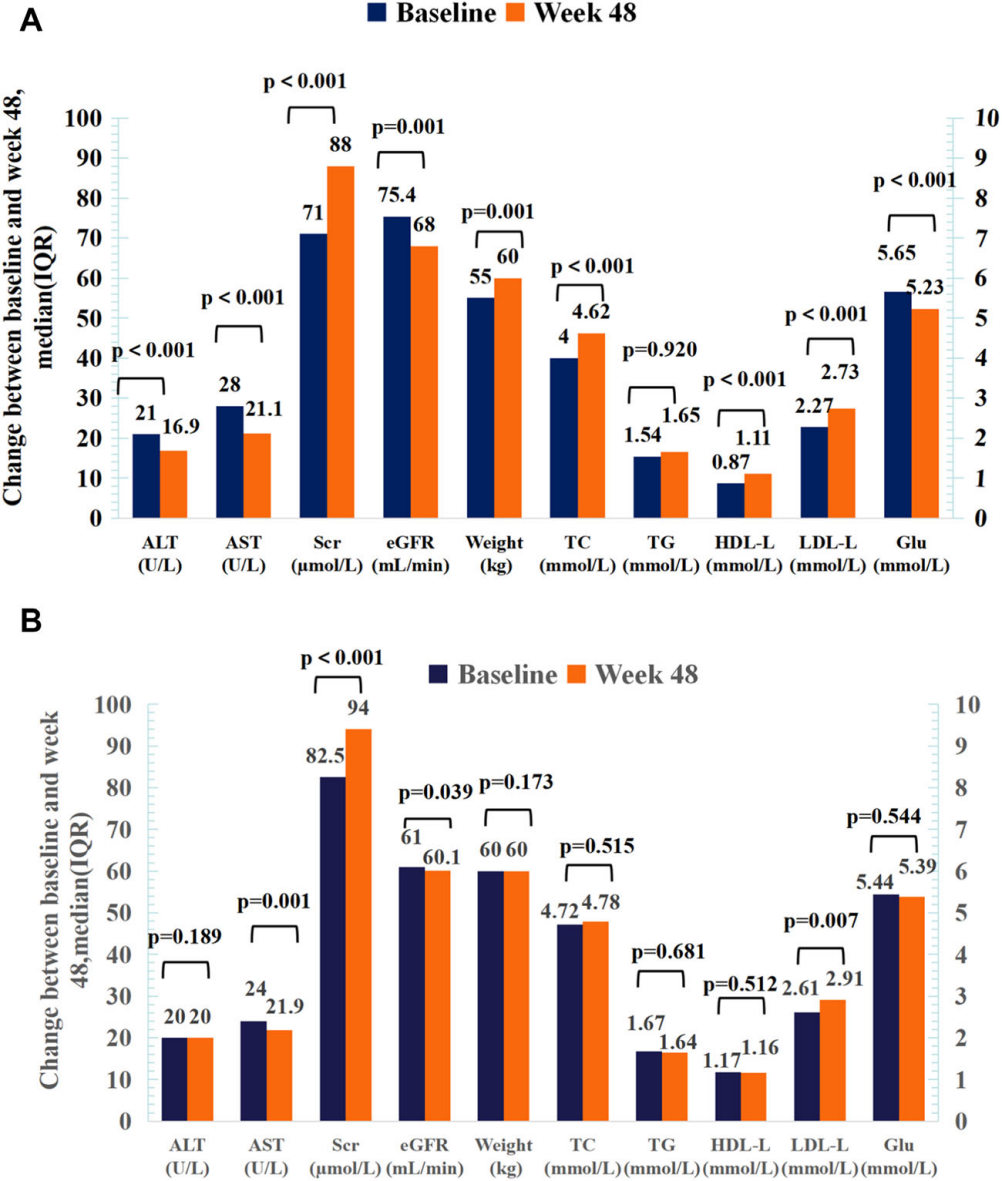
Changes in biochemical indices between baseline and Week 48. (A) Body weight, immunological response, and biochemical indices of ART‐naïve patients after using the B/F/TAF regimen. (B) Body weight, immunological response, and biochemical indices of ART‐experienced patients after using the B/F/TAF regimen. Data are presented as median (interquartile range). Abbreviations: ALT, alanine aminotransferase; AST, aspartate aminotransferase; CKD‐EPI Scr, Chronic Kidney Disease Epidemiology Collaboration value calculated based on serum creatinine concentration; Glu, glucose (blood); HDL‐C, high‐density lipoprotein cholesterol; LDL‐C, low‐density lipoprotein cholesterol; Scr, serum creatinine; UA, uric acid; TC, total cholesterol; TG, triglyceride.

After 48 weeks of treatment with the B/F/TAF regimen, the body weights and biochemical indexes of the patients were obtained (Figure 3A). Among the 145 ART‐naïve patients, ALT (21.0 vs. 16.9 U/L, *p* < 0.001), AST (28.0 vs. 21.1 U/L, *p* < 0.001), eGFR (75.4 vs. 68.0 U/L, *p* < 0.001) were significantly reduced at week 48 compared to baseline. Meanwhile, weight (55.0 vs. 60.0 kg, *p* = 0.001), Scr (71.0 vs. 88.0 U/L, *p* < 0.001), TC (4.0 vs. 4.62 U/L, *p* < 0.001), HDL (0.87 vs. 1.11 U/L, *p* < 0.001) and LDL (2.27 vs. 2.73 U/L, *p* < 0.001) were significantly elevated. In the 141 ART‐experienced cases, eGFR was significantly increased at week 48 compared to baseline (*p* < 0.05), while AST, LDL, and Scr were significantly decreased (*p* < 0.05); the remaining indicators showed no significant changes versus baseline (*p* > 0.05) (Figure 3B).

### 3.5 Adverse Events

The ART‐naive and ART‐experienced groups displayed 17 (11.7%) and 6 (4.3%) adverse effects, respectively. The most common adverse events in the ART‐naive group were rash (3.4%), osteopenia (3.4%), dizziness (2 cases, 1.4%), and leg weakness (1.4%). Serious adverse events were found in 4 cases (11.7%). In the ART‐experienced group, the most common adverse events were insomnia (1.4%), osteopenia (1.4%), and joint pain (0.7%). The incidence of drug‐related adverse events was 0.7% (Table 3).

**Table 3:** Overview of adverse events associated with B/F/TAF in the study cohort.

Adverse events	ART‐naïve (145)	ART‐experienced (141)
All adverse events	17 (11.7%)	6 (4.3%)
Rash	5 (3.4%)	0 (0)
Dizziness	2 (1.4%)	0 (0)
lose sleep	1 (0.7%)	2 (1.4%)
Bone loss	5 (3.4%)	2 (1.4%)
Skelasthenia	2 (1.4%)	0 (0)
Abdominal pain	1 (0.7%)	0 (0)
Arthralgia	0 (0)	1(0.7%)
Diminution of vision	1 (0.7%)	0 (0)
Serious adverse events	4 (11.7%)	1 (0.7%)
Drug‐related adverse event		
Herpes	1 (0.7%)	0 (0)
Abundant dreams	0 (0)	1 (0.7%)

## 4 Discussion

Many previous studies have focused on comparing immune and therapeutic effects between adult patients initially administered B/F/TAF and other INSTI [14, 15, 16]. There is no centralized study on the elderly, and this study is the first in southwest China to investigate the clinical effectiveness and safety of B/F/TAF in elderly patients. In this retrospective study, 44.3% of patients receiving ART for the first time were diagnosed with advanced disease (CD4 < 200/μL) at admission, 25.5% had baseline HIV‐1 RNA ≥ 500,000 copies/mL, 34.9% had severe AIDS‐related infections, and over 44% were on additional medications. Despite these factors, a high viral suppression rate (90.6%) was observed at 48 weeks, consistent with previous reports examining young patients treated with B/F/TAF [17, 18]. This indicates that despite the high drug load and various comorbidities in elderly patients, there is still a good virological response. In ART‐experienced patients, the primary reason for switching was to optimize treatment (34.2%). After 48 weeks of treatment, 92.9% of patients achieved viral suppression, which is similar to the virological suppression rate reported by Shi Yuzhi in their study comparing other regimen switches to B/F/TAF [17].

The HIV virus mainly attacks human immune cells, and the lower the CD4 + T lymphocyte count, the higher the incidence of adverse outcomes, including opportunistic infections, non‐AIDS complications (e.g., metabolic syndrome, cardiovascular disease, osteoporosis and fractures, liver disease, kidney disease, and HIV‐related neurocognitive dysfunction) and death [19]. Therefore, monitoring CD4+ cell count and CD4 + /CD8+ cell ratio can comprehensively reflect the body's immune status and evaluate immune recovery. According to our results, after 48 weeks of treatment, the CD4+ cell count and CD4 + /CD8+ cell ratio in both the “initial use” and “switch” groups showed a significant increase from baseline, consistent with previous studies [18, 20], and both observed a better immunological response. This study found that CD4+ cell count and CD4 + /CD8+ cell ratio in the ART‐naive group increased significantly compared with the “experienced” group at baseline, which may be due to the fact that the CD4+ cell count of the ART‐naive group was < 200/μL at the time of enrollment, and the immune function recovered quickly after receiving antiviral treatment.

The study highlighted that metabolic abnormalities are common problems during treatment with B/F/TAF [21]. Abnormal lipid levels, characterized by elevated levels of low‐density lipoprotein cholesterol or total cholesterol, are important risk factors for atherosclerosis and cardiovascular disease, and occur frequently in HIV‐infected patients, especially elderly cases [13]. Clinicians should pay more attention to the lipid level of elderly patients. Our results showed that after 48 weeks of treatment, total cholesterol, low‐density lipoprotein cholesterol and high‐density lipoprotein cholesterol in the ART‐naive group were significantly increased from baseline, similar to previous studies [22]. In this cohort, switching to B/F/TAF did not lead to significant reductions in total cholesterol, low‐density lipoprotein cholesterol, and triglycerides, corroborating findings by Mazzitelli et al [23]. Previous studies have also pointed out that NRTIs and NNRTIs usually increase TC, LDL‐C, HDL‐C and TG levels, although the exact mechanism is unclear [24]. Therefore, when using BIC/TAF/FTC in elderly patients, changes in lipid profile should be closely monitored. More interestingly, after 48 weeks of B/F/TAF treatment, the weights of patients with initial ART significantly increased compared to baseline (55.80 kg vs. 60 kg, *p* < 0.01). A study by Gan Lin [18]. in China found that after 48 weeks of initial B/F/TAF treatment, the patients’ weights increased significantly by 4.05 kg averagely, which corroborates this study. As shown above, 34.9% of patients who started treatment had opportunist infections at baseline and advanced AIDS, and in HIV‐1 patients, recovery of health may lead to weight gain. As patients age, they lose glomerular function, which is worth paying attention to. In the “naïve” and “experienced” groups, renal function was significantly improved after treatment, characterized by a decrease in SCr levels and an increase in eGFR. Ultimately, we found no evidence of adverse kidney events, and no patients discontinued the medication due to impaired renal function. This indicates that B/F/TAF, which contains TAF, is safe for the kidney, consistent with previously reported 5‐year data on B/F/TAF [22].

Initial use of B/F/TAF and conversion to B/F/TAF with virologic suppression proved to be effective, even in the presence of multiple comorbidities and other concomitant drugs. This study had some limitations and bias. First, due to the retrospective nature of this study, many confounding factors could not be controlled. This study may have only included patients with complete medical records, while those lost to follow‐up were excluded, potentially leading to an overestimation of treatment efficacy. Second, this was a single‐center study with a follow‐up period of only 48 weeks, and the patients were elderly, which may limit the generalizability of the findings. Future studies could examine additional populations for continued observation up to 96 weeks, 144 weeks, etc., to obtain more reliable data on older adults.

## 5 Conclusions

The current study suggests that B/F/TAF is a friendly option for elderly HIV patients to achieve and maintain virological suppression and immune recovery. Although dyslipidemia may occur in patients initially administered the B/F/TAF regimen, this may be due to aging, as comorbidities and the need for combination therapy are more common in elderly populations, and it is important to collect data in prolonged follow‐up in elderly PLWH to optimize overall health and well‐being.

## Author Contributions

**Shujing Ma:** conceptualization, writing – original draft. **Xiaoxin Xie:** conceptualization, data curation. **Yanhua Fu:** conceptualization, investigation. **Lin Gan:** methodology. **Xiaoyan Yang:** investigation. **Qing Wang:** investigation. **Hai Long:** formal analysis, methodology, supervision.

## Ethics Statement

The Ethics Committee of Guiyang Public Health Clinical Center (202226) approved this study.

## Consent

The informed consent requirement was waived since the study was retrospective. This study complied with the Declaration of Helsinki.

## Conflicts of Interest

The authors have no relevant affiliations or financial involvement with any organization or entity with a financial interest in or financial conflict with the subject matter or materials discussed in the manuscript. This includes employment, consultancies, honoraria, stock ownership or options, expert testimony, grants or patents received or pending, or royalties.

## Data Availability

The datasets used or analysed during the current study available from the corresponding author on reasonable request.
